# Alteration of intracellular protein expressions as a key mechanism of the deterioration of bacterial denitrification caused by copper oxide nanoparticles

**DOI:** 10.1038/srep15824

**Published:** 2015-10-28

**Authors:** Yinglong Su, Xiong Zheng, Yinguang Chen, Mu Li, Kun Liu

**Affiliations:** 1 State Key Laboratory of Pollution Control and Resource Reuse, School of Environmental Science and Engineering, Tongji University, 1239 Siping Road, Shanghai 200092, China

## Abstract

The increasing production and utilization of copper oxide nanoparticles (CuO NPs) result in the releases into the environment. However, the influence of CuO NPs on bacterial denitrification, one of the most important pathways to transform nitrate to dinitrogen in environment, has seldom been studied. Here we reported that CuO NPs caused a significant alteration of key protein expressions of a model denitrifier, *Paracoccus denitrificans*, leading to severe inhibition to denitrification. Total nitrogen removal efficiency was decreased from 98.3% to 62.1% with the increase of CuO NPs from 0.05 to 0.25 mg/L. Cellular morphology and integrity studies indicated that nanoparticles entered the cells. The proteomic bioinformatics analysis showed that CuO NPs caused regulation of proteins involved in nitrogen metabolism, electron transfer and substance transport. The down-regulation of GtsB protein (responsible for glucose transport) decreased the production of NADH (electron donor for denitrification). Also, the expressions of key electron-transfer proteins (including NADH dehydrogenase and cytochrome) were suppressed by CuO NPs, which adversely affected electrons transfer for denitrification. Further investigation revealed that CuO NPs significantly inhibited the expressions and catalytic activities of nitrate reductase and nitrite reductase. These results provided a fundamental understanding of the negative influences of CuO NPs on bacterial denitrification.

Nitrogen cycle, one of the primary biogeochemical cycles in biosphere, accomplishes the transformation of nitrogen in different forms among atmosphere, water, land and organisms. Denitrification process, which converts nitrate to dinitrogen and returns nitrogen element to the atmosphere, is of great significance for the close relation to global climate change, water quality and ecosystem health^1^. As the most widely distributed performer of denitrification, denitrifying bacteria accomplish the reduction of nitrate via a series of biological processes. The denitrifying metabolic reactions are ultimately controlled by various functional proteins, such as electron transfer proteins and denitrifying enzymes. It was reported that the expressions of these proteins determined the cellular metabolism of denitrifiers^2^,^3^.

With the rapid development of nanotechnology, engineered nanomaterials have been widely applied in various fields, such as biomedicine, material synthesis, and chemical catalysis^4^,^5^. It has been reported that nanomaterials could pose negative effects on human cells^6^,^7^,^8^, plants^9^ and model bacteria^10^. In particular, due to the outstanding antimicrobial property, copper oxide nanoparticles (CuO NPs) have been widely applied in antimicrobial textiles, wood preservation, antifouling paints, and agricultural biocides^11^,^12^. It was reported that the global production of CuO NPs was 570 tons in 2014, and the estimated production would be 1600 tons by the year 2025^13^. Hence, the increasing production and utilization of CuO NPs result in their intentional or unintentional releases into the environment^14^. Although the toxicity of CuO NPs to some model organisms, such as human cells or algae, had been widely studied^15^,^16^, the main reason was attributed to the generation of reactive oxygen species (ROS)^4^, which might lead to the DNA lesion or gene regulation^9^,^15^. It is well-known that proteins, the final products of gene expression, are the terminal executors of specific biological processes, such as catalyzing metabolic reactions and transporting substances. Therefore, once the protein expressions were significantly affected, the growth and global metabolic processes would be altered^17^.

During denitrification the extracellular carbon source (such as glucose) need to be transported into cells before being utilized for microbial growth and denitrification^18^,^19^,^20^. It has been well documented that the transport process of glucose is accomplished by the cooperation of several important proteins, such as solute-binding protein (GtsA), inner membrane protein (GtsB and GtsC), and ATP-binding protein (MalK)^21^. Moreover, bacterial denitrification is a series of sequential redox reactions relying on electron transfer. In the electron transfer chain, electrons are sequentially delivered by electron transfer proteins such as cytochrome bc1, cytochrome c, and denitrifying enzymes^22^. In addition, four key denitrifying enzymes, nitrate reductase (NAR), nitrite reductase (NIR), nitric oxide reductase (NOR) and nitrous oxide reductase (N_2_OR), sequentially catalyze the reduction reactions from nitrate to nitrogen. Previous studies indicated that the changes of key electron transport proteins or denitrifying enzymes affected the denitrification performance^23^,^24^,^25^. However, the influences of engineered nanomaterials on denitrifying bacterial intracellular functional protein expressions and functions have seldom been documented.

In this study, the potential effects of CuO NPs on denitrification were investigated and the mechanisms were explored from the aspect of regulation of intracellular protein expressions. *Paracoccus denitrificans* is widely found in soil, water and sediments^26^, and can carry out sequential reduction of nitrate, nitrite, nitric oxide and nitrous oxide to dinitrogen gas^27^. Thus, *P. denitrificans* has been recognized as an excellent model denitrifying bacterium to determine the effects on biological denitrification^28^. The influences of CuO NPs on cellular morphology and structure integrity were studied by transmission electron microscope (TEM) and lactate dehydrogenase (LDH) release assays. Isobaric tags for relative and absolute quantitation (iTRAQ) technique provided the overall proteome information, and Gene Ontology (GO) and Kyoto Encyclopedia of Genes and Genomes (KEGG) analyses classified the differentially expressed proteins into cellular functions and processes. The regulation changes in intracellular proteins involved in some vital functions closely related to denitrification were further confirmed by multiple reaction monitoring (MRM) quantification.

## Results and Discussion

### Effects of CuO NPs on bacterial denitrification performance

**Figure 1:**
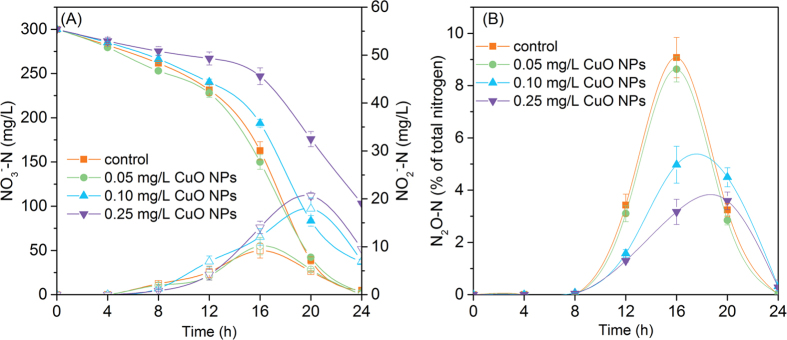
Effects of CuO NPs on the variations of NO_3_^−^-N (solid, A), NO_2_^−^-N (hollow, A) and N_2_O-N (B) during 24 h exposure tests. Error bars represent standard deviations of triplicate measurements.

Denitrifying bacteria (such as *Paracoccus denitrificans* in this study) employ carbon source (such as glucose) and nitrate respectively as electron donor and electron acceptor to accomplish the denitrification process under anaerobic circumstance, in which nitrate is reduced step by step to nitrite, nitric oxide, nitrous oxide, and finally nitrogen^22^. In this study, the effects of CuO NPs on the variations of NO_3_^−^, NO_2_^−^ and N_2_O are shown in Fig. 1. In the control (without the presence of CuO NPs), nitrate was reduced rapidly and the final nitrate removal efficiency was 98.4%. In the presence of 0.05 mg/L CuO NPs, the nitrate removal efficiency was 99.1%, which showed no significant difference with that in the control. However, with the increment of CuO NPs to 0.10 and 0.25 mg/L, the nitrate removal efficiency was decreased to 87.7% and 65.6%, respectively.

Although a bit of transient accumulation of nitrite was observed during the denitrification process, there was no detectable nitrite at 24 h in the absence (the control) and presence of 0.05 mg/L CuO NPs. The final nitrite concentration, however, was 6.94 and 9.54 mg/L at CuO NPs of 0.10 and 0.25 mg/L, respectively. From Fig. 1B it can be seen that the maximal N_2_O accumulation was decreased with the increase of CuO NPs, but there was no detectable N_2_O appeared by the end of experiments no matter whether CuO NPs were present or not. Thus, the data of this study showed that the presence of CuO NPs led to a lower efficiency of nitrate reduction, and caused higher nitrite accumulation and less N_2_O emission during denitrification. In the following text, the reasons for CuO NPs inhibiting denitrification were explored.

### Interaction between CuO NPs and bacterial cells

Usually, the toxicity of metal oxide nanoparticles was attributed to the release of ion^29^,^30^ or the small size of nanoparticles^31^. Therefore, the dissolved Cu^2+^ from CuO NPs in mineral media was measured, and the effects of copper ion control on *P. denitrificans* were investigated. The dissolution data showed that the dissolved ion concentration was dependent on NPs dose and time (Figure S1A, Supplementary information). In detail, after 24 h dissolution, 0.0069, 0.0115 and 0.0149 mg/L Cu^2+^ were detected in the media for 0.05, 0.1 and 0.25 mg/L CuO NPs, and the corresponding dissolution ratios were 13.82%, 11.51% and 5.96% respectively. Then, the results of ion toxicity test in Figure S1B (in Supplementary information) indicated that the presence of Cu^2+^ in the range of 0.0069 (dissolved from 0.05 mg/L CuO NPs) to 0.0149 mg/L (dissolved from 0.25 mg/L CuO NPs) caused insignificant effects on the cell viability of *P. denitrificans* (*p* > 0.05). Also, the denitrification processes of *P. denitrificans* with or without the presence of copper ion were investigated (Figure S1C and Figure S1D in Supplementary information), and the Cu^2+^ did not cause significant effects on the reductions of NO_3_^−^-N and NO_2_^−^-N and the final N_2_O concentration. Likewise, the presence of Cu^2+^ did not inhibit the catalytic activity of denitrifying enzymes (Figure S2 in Supplementary information). Therefore, Cu^2+^ did not account for the severe influence of CuO NPs on *P. denitrificans*, and similar conclusions were made in the literatures^9^,^16^. In the coming text, the toxic mechanism was developed from the interaction between CuO NPs and bacteria.

**Figure 2:**
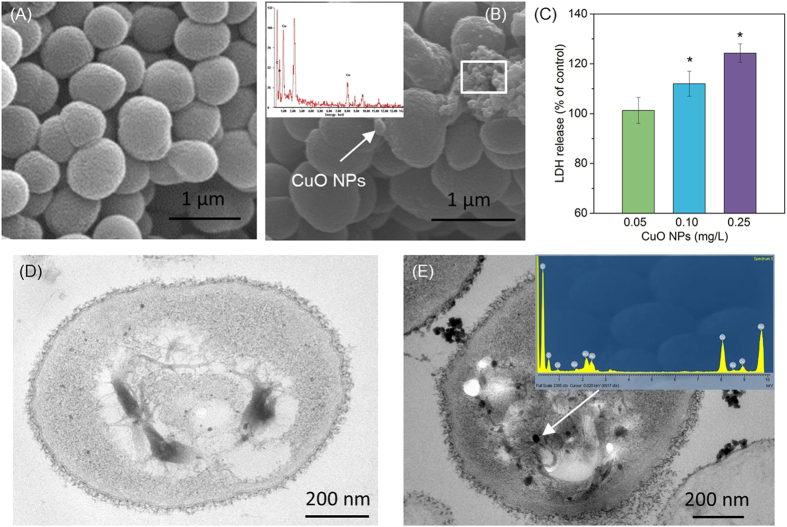
Effect of CuO NPs on cellular structure of *P. denitrificans*. SEM image of bacteria (**A**), SEM image and EDS analysis of bacteria exposed to 0.25 mg/L CuO NPs (**B**), LDH release of bacteria exposed to different CuO NPs dose (**C**), TEM image of bacterial cell without CuO NPs exposure (**D**), and TEM image and EDS analysis of cells exposed to 0.25 mg/L CuO NPs (**E**).

Previous studies have reported that some nanoparticles (such as TiO_2_ NPs) can easily attach to cell surface due to their adsorption property, which results in their toxic effects^32^,^33^. In this study, the scanning electron microscope (SEM) images with energy dispersive spectroscopy (EDS) analysis showed that CuO NPs could adsorb on the surface of *P. denitrificans* (Fig. 2B). The data in Fig. 2C showed that the presence of CuO NPs caused the LDH release of *P. denitrificans*, and the level of released LDH increased with increasing the CuO NPs concentrations. Generally, LDH is a stable enzyme presenting in cytoplasm, and its release is used as a marker for the membrane leakage because the intracellular LDH would be released into the cell culture upon damage of cell membrane^31^. In this study, the LDH variation induced by CuO NPs exposure indicated that CuO NPs affected the bacterial membrane integrity and caused membrane damage. Also, it was observed that the growth and viability of *P. denitrificans* were inhibited (Figure S3, Supplementary information). Transmission electron microscope-energy dispersive spectrometer (TEM-EDS) analysis revealed that there were CuO NPs inside *P. denitrificans* (Fig. 2E), which suggested that nanoparticles entered the cells.

It is well-known that large amounts of proteins in bacteria are responsible for various biological functions. For example, the uptake of carbon source and trace elements from extracellular circumstance relies on transporter proteins^21^,^34^. To denitrifying bacteria, the denitrification reaction greatly depends on the proteins involved in electron transfer chain. As CuO NPs have been inside *P. denitrificans* and the cellular metabolism is dependent on the fate of proteins, proteomic method based on MS technique was therefore adopted in the following text to explore the underlying mechanisms of CuO NPs affecting denitrification.

### Overall analysis of proteomic data in the absence and presence of CuO NP

The iTRAQ analysis data showed that 7976 unique peptides and 1449 proteins were identified. Compared with the control (cells without CuO NPs exposure), there were 138 differentially expressed proteins (62 down-regulated and 76 up-regulated) after exposure to 0.25 mg/L CuO NPs for 24 h. Since Gene Ontology (GO) annotation could provide the important information of proteins involved in biological process, cellular component, and molecular function^35^, in this study the identified 138 differentially expressed proteins were assigned to the GO terms. Figure S4 (Supplementary information) showed that the differentially expressed proteins were mainly related to the catalytic activity, binding, and transporter activity at the level of molecular function. Based on the metabolic processes in which various proteins were involved, the results of Kyoto Encyclopedia of Genes and Genomes (KEGG) annotation indicated that most of differentially expressed proteins induced by CuO NPs were classified to ATP-binding cassette (ABC) transporter (Figure S5, Supplementary information). The proteins related to this process were one of the largest protein families with diverse physiological functions. Apparently, the presence of CuO NPs influenced cellular metabolic process such as catalytic reactions and transport.

**Figure 3:**
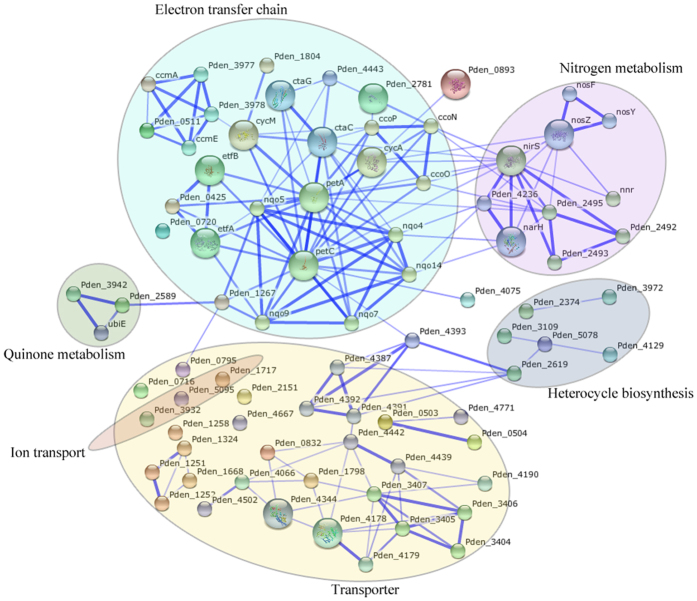
The interaction network of differential proteins induced by CuO NPs. The network was created by the STRING algorithm, and strong interactions are represented by thicker lines.

For a better understanding of the underlying cellular responses triggered by CuO NPs, STRING (Search Tool for the Retrieval of Interacting Genes/Proteins) algorithm^36^ was used to build protein-protein interaction networks. The differentially expressed proteins induced by 0.25 mg/L CuO NPs were shown in Fig. 3, and the lines depicted the interacting relations of various proteins. The proteins were identified and classified by specific biological functions, and it was clear that the nitrogen metabolism, electron transfer and transport were three predominant functions being affected for most regulated differential proteins belonged to these categories. For denitrifying bacteria, denitrification process relied on nitrogen metabolism, so the affected expressions of proteins in this category might cause direct influence on denitrification performance. Moreover, the transformation from nitrate to dinitrogen was a series of sequential reduction reactions, which required the involvement of electron transfer. It is reasonable that electron transfer was closely associated with nitrogen transformation, and this strong interaction was also observed in the network (Fig. 3). In addition, transport proteins, playing the roles of transporting substance (such as nutrient substance and ion) for growth and denitrification, exhibited differential expressions under CuO NPs stress. Therefore, CuO NPs exposure induced the regulation of nitrogen metabolic proteins, electron transfer proteins and transport proteins, which would disturb the denitrification directly or indirectly.

### Effects of CuO NPs on substance transmembrane transport and intracellular transformation

**Figure 4:**
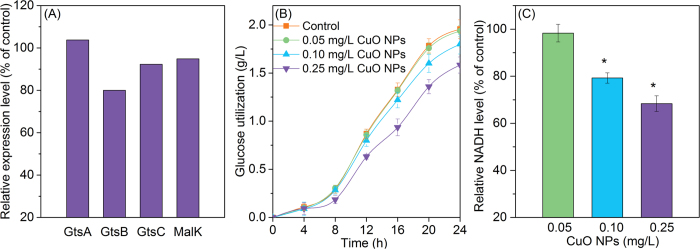
Relative expressions of glucose transporter proteins of denitrifying bacteria (A), glucose utilization in the absence and presence of CuO NPs (B), and relative intracellular NADH level of *P. denitrificans* after exposure to CuO NPs (C).

Figure S6 (Supplementary information) illustrates that glucose (carbon source) is firstly transported into bacteria by carbohydrate ABC transporter membrane proteins including extracellular solute-binding protein (GtsA), binding-protein-dependent transport system inner membrane protein (GtsB, GtsC), and transport system ATP-binding protein (MalK)^34^. Then, the intracellular glucose goes through glycolysis process and is degraded with the generation of NADH (the direct electron donor) for subsequent denitrification. Figure 4A reveals that the expression level of GtsB was 80% of the control, and the down-regulation was verified by the MRM quantification. Figure 4B further shows that the utilization of glucose was decreased with the increase of CuO NPs dosage, i.e., from 1.96 (control) to 1.80 (0.10 mg/L CuO NPs) and 1.58 g/L (0.25 mg/L CuO NPs). After glucose is transported into bacteria, it is degraded and NADH is generated. It can be seen from Figure S7 (Supplementary information) that the expressions of enzymes involved in glycolysis process were unaffected, suggesting that the CuO NPs exposure did not disturb glucose degradation. However, the data in Fig. 4C indicated that the intracellular NADH level was decreased significantly at CuO NPs of over 0.05 mg/L, and the inhibitory effect was magnified with the increase of dosage. Therefore, the inhibition to glucose transport proteins led to less available glucose for bacterial intracellular metabolism, which further attributed to the decreasing of NADH generation.

**Figure 5:**
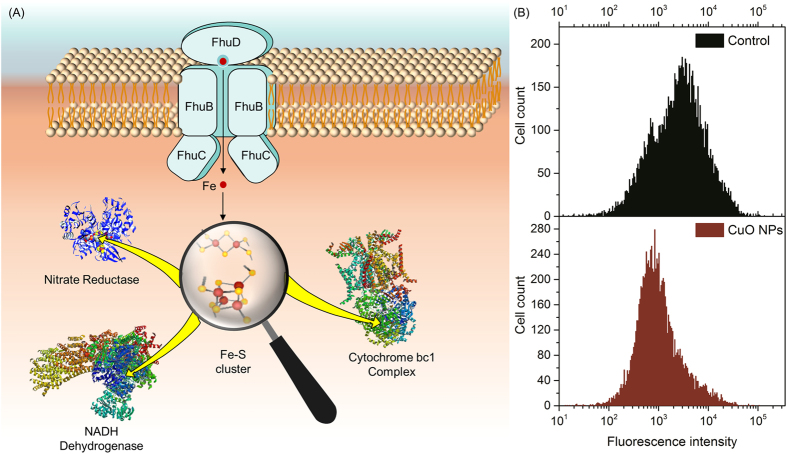
Illustration of the iron transport and its roles in Fe-S proteins synthesis in *P. denitrificans* (A), the fluorescence intensity distribution of iron-binding probe in bacteria without CuO NPs treatment (the up panel) and exposed to 0.25 mg/L CuO NPs (the down panel) by flow cytometry analysis (B).

In denitrification process, large amounts of metalloproteins (such as cytochrome, dehydrogenase and reductase) contain iron atom, which acts as the active center for redox reactions. It has been reported that *P. denitrificans* cells acquire iron for metalloproteins synthesis from extracellular circumstance via ferric hydroxamate uptake (Fhu) proteins^37^, which consists of substrate-binding protein FhuD, permease protein FhuB and ATP-binding protein FhuC (Fig. 5A). Because the synthesis of vital iron-contained proteins depended on cellular iron transport^38^, the influence of CuO NPs on the expressions of iron transport proteins were investigated. The expression level of protein FhuD under 0.25 mg/L CuO NPs stress was 60.6% of the control, indicating that CuO NPs caused significant down-regulation of iron transport protein. The iron uptake inhibition was further verified by the intracellular iron quantification, and CuO NPs significantly reduced intracellular available iron for the mean fluorescence intensity emitted by iron-binding probe decreased from 4660 (the control) to 1903 (at the presence of 0.25 mg/L CuO NPs) in Fig. 5B.

### Effects of CuO NPs on the electron transfer and denitrification reactions

**Figure 6:**
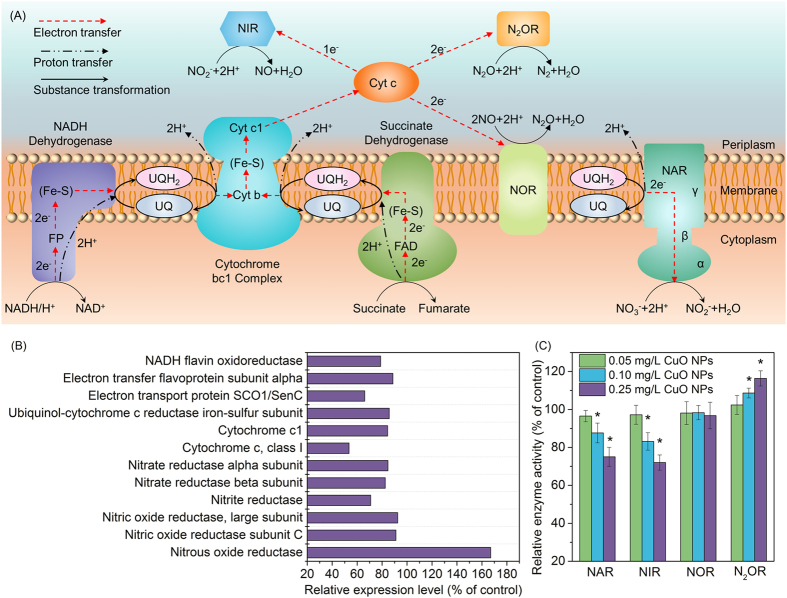
Illustration of electron transfer and substance transformation in denitrification process (A), the relative expression level of proteins involved in denitrification (B), the effects of CuO NPs exposure on the activities of NAR, NIR, NOR, and N2OR at time of 24 h (C). Error bars represent standard deviations of triplicate measurements, and asterisks indicate significant reduction compared to control samples (*p* < 0.05).

As illustrated in Fig. 6A, in the electron transfer process electron donors are catalyzed by NADH dehydrogenase and succinate dehydrogenase, and the generated electrons are delivered to ubiquinone, cytochrome bc1 complex, and finally transferred to key denitrifying enzymes via cytochrome c^22^,^39^. For a deeply understanding of CuO NPs affecting on denitrification, the fate of proteins involved in electron transfer process was investigated. It can be seen from Fig. 6B that the presence of CuO NPs significantly affected several proteins in electron transfer chain. The expressions of two subunits of NADH dehydrogenase (i.e., NADH flavin oxidoreductase and electron transfer flavoprotein subunit alpha) in the presence of 0.25 mg/L CuO NPs were respectively 78.8% and 88.6% of the control. The expressions of cytochrome c, cytochrome c1, ubiquinol-cytochrome c reductase iron-sulfur subunit, and SCO1/SenC were 53.5%, 84.4%, 85.7% and 66% of the control respectively, which all showed down-regulation when exposed to CuO NPs. Clearly, the presence of CuO NPs induced the down-regulated expressions of key proteins involved in electron transfer chain, which resulted in low electron transfer efficiency for subsequent denitrification reactions.

After being transferred by electron carrier, electrons are delivered to specific denitrifying enzymes for reduction reactions. In denitrification reactions, NAR undertakes the function to reduce nitrate, and this enzyme comprises three subunits (α, β and γ)^40^. Electrons are transferred from γ-subunit to β-subunit and finally α-subunit, the active site for nitrate reduction. Figure 6B depicted that both the expressions of α-subunit and β-subunit of NAR were decreased to 84.7% and 82.5% of the control in the presence of CuO NPs, and the enzymatic viability tests confirmed the lower catalytic activity of NAR induced by CuO NPs (Fig. 6C). Meanwhile, the expression of nitrite reductase was down-regulated by 29.2%, and similar decreasing pattern of NIR activity was shown in Fig. 6C. These results were in correspondence with the observations of CuO NPs inducing more accumulation of nitrate and nitrite. Moreover, CuO NPs induced the up-regulated expression (167%) and elevated the activity of nitrous oxide reductase, which was attributed to the release of trace Cu^2+^ (0.016 mg/L) from 0.25 mg/L CuO NPs, as its presence was reported to be in favor of the catalytic activity of N_2_OR^41^,^42^. These data accounted for the phenomenon that the maximum accumulation of N_2_O descended with the increment of CuO NPs dosage (Fig. 1B).

In summary, the presence of CuO NPs could cause significant effects on the denitrification function of *P. denitrificans*, such as less nitrate reduction, more nitrite accumulation and less nitrous oxide maximum accumulation. The damaged membrane led to the entry of CuO NPs into bacteria, and then the proteomic alteration was induced by intracellular CuO NPs. Bioinformatic analysis indicated that some vital metabolic functions or pathways, such as substance transportation, electron transfer, and catalytic activity, were affected by CuO NPs significantly. Specifically, the glucose transport proteins were down-regulated, leading to less electron donor NADH for denitrification. Also, the restriction towards iron uptake resulted in less available iron, which was adverse for the synthesis and function of iron-contained proteins in denitrification process. In the electron transfer chain, several important electron transfer proteins and denitrifying enzymes were also affected by CuO NPs, and enzyme activity measurements accorded with the down-regulation of NAR and NIR and up-regulation of N_2_OR. All these observations were in correspondence with the decreased denitrification performance induced by CuO NPs.

## Methods

### Nanoparticles preparation and characterization

CuO NPs (particle size < 50 nm) were purchased from Sigma-Aldrich (St. Louis, MO). X-ray diffraction (XRD) analysis was conducted on a Bruker D8 Advance diffractometer with a Cu Kα radiation source and the result is shown in Figure S8 (Supplementary information). The powder CuO NPs were ultrasonicated in alcohol and then dropped into Ni grids for TEM analysis (Figure S9, Supplementary information). Also, the 50 mg/L NPs stock suspension was prepared by dispersing 0.05 g NPs powder in 1 L Milli-Q water, which was followed by 1 h ultrasonication (25 °C, 500 W, 40 kHz). The size distribution of particles in stock suspension was determined by laser diffraction technique via a Mastersizer 3000 (Malvern UK).

### Cultivations of denitrifying bacteria

The strain of *Paracoccus denitrificans* (ATCC 19367) is widely appearing in aquatic and soil environments and the metabolic mechanism is well understood for research^22^. In this study, *Paracoccus denitrificans* was employed as the test denitrifying microbe, which was purchased from American Type Culture Collection. Before the exposure to CuO NPs, *P. denitrificans* was cultivated aerobically in Difco nutrient broth at 30 °C in a shaker with constant agitation (200 rpm) for 24 h. Then, the bacteria were harvested from broth media for the following exposure tests.

### Experiments of denitrifying bacteria exposure to CuO NPs

Firstly, mineral medium was prepared in serum bottles, and the composition was from the reference with minor modification (g/L): glucose, 5.0; K_2_HPO_4_, 7.0; KH_2_PO_4_, 3.0; sodium citrate·2H_2_O, 0.5; MgSO_4_·7H_2_O, 0.1; (NH_4_)_2_SO_4_, 1.0; KNO_3_, 2.16 and trace elements solution of 50 μL/L^28^. The trace elements solution contained (g/L): CaCl_2_·2H_2_O, 7.4; MnSO_4_·H_2_O, 1.0; ZnSO_4_·7H_2_O, 3.6; CoCl_2_·6H_2_O, 0.4; FeCl_3_·6H_2_O, 0.96; CuCl_2_·2H_2_O, 0.03; H_3_BO_3_, 0.3; NiCl_2_·6H_2_O, 0.01; EDTA-Na_2_, 3.7. According to the reference, ammonium sulfate was used to preclude assimilatory nitrate reduction and ensure that nitrate consumption was caused by respiration^23^. All serum bottles and correlative equipments were sterilized in a high-pressure steam sterilization pot and then disinfected by ultraviolet rays for 20 min. In an anaerobic chamber, CuO NPs stock suspension was added to the mineral medium, and then bacterial cultures were inoculated at an initial optical density (OD_600_) value of 0.05. After well-prepared, the serum bottles were placed in a shaker (200 rpm) with constant temperature of 30 ± 1 °C, and the samples were taken for analysis every 4 h.

### Protein extraction, digestion and iTRAQ labeling

After exposed to 0 or 0.25 mg/L CuO NPs for 24 h, triplicated samples were obtained from each serum bottle, and then mixed for iTRAQ test. The bacteria pellets were obtained by centrifugation at 5000 rpm firstly. After the addition of prepared STD buffer (containing 4% sodium dodecyl sulfate (SDS) and 150 mM Tris-HCl (pH 8.0)), the samples were treated by vortex mixing, heating in water batch for 5 min, and sonic disruption (80 W, 10 s operation and 15 s break for 10 cycles). Finally, the supernatant was collected by centrifugation (4 °C, 15000 rpm, 10 min) after water batch heating, and protein concentration was determined by the method of Lowry *et al.*^43^.

The extracted proteins were digested according to the reference^44^. Briefly, 400 μg protein of each sample was diluted in 30 μL STD buffer (4% SDS, 100 mM Tris-HCl (pH 8.0), and 100 mM dithiotreitol). Then, the solution was incubated in boiling water for 5 min, cooled down to room temperature, diluted with 200 μL UA buffer (8 M urea, 150 mM Tris-HCl (pH 8.0)), and transferred into a 10 kD filter for ultrafiltration. The samples were centrifuged at 14000 *g* for 15 min (4 °C), added with 200 μL UA buffer, and centrifuged again. After adding 100 μL iodoacetamide (50 mM in UA buffer), samples were placed in darkness for 20 min min, centrifuged under the above conditions, and washed with UA buffer for twice. 10 μL DS buffer (50 mM triethylammonium bicarbonate, pH 8.5) was added to the samples, and then centrifuged (14000*g* for 15 min at 4 °C). This operation was repeated for twice. Finally, 40 μL trypsin (5 μg trypsin in 40 μL DS buffer) was added, and the samples were incubated at 37 °C for 16 h. The resulting peptides were collected in new tubes before centrifugation, and the peptide content was measured at 280 nm.

After digestion, 80 μg peptide was labeled with iTRAQ reagents according to the instructions of manufacturer (Applied Biosystems, USA). The samples #1 (without CuO NPs exposure) and #2 (with 0.25 mg/L CuO NPs exposure) were respectively reacted with reagent 114 and 115, and then the labeled samples were mixed and 1/5 of mixture was taken to desalt by C_18_Cartridge (Sigma) before being dried in vacuum.

### Liquid chromatography (LC) separation and mass spectra (MS) quantification of peptides

The separation process was performed by nanopump High Performance Liquid Chromatography (HPLC) system Easy nLC. The column was equilibrated with 95% (v/v) Buffer A (0.1% (v/v) formic acid), and then the samples were injected into two Thermo scientific EASY column columns (2 cm × 100 μm 5 μm-C18; 75 μm × 100 mm 3 μm-C18) successively with the flow rate of 250 nL/min. Peptides were separated with Buffer B (0.1% formic acid and 84% acetonitrile in MilliQ water) according to following segmented gradient: 0–55% for 220 min, 55%–100% for 8 min, and finally 100% for 12 min.

After separation, samples were analyzed by Q-Exactive (Thermo Finnigan, USA) mass spectrometer (with a mass range of 300–1800 *m/z*) in the positive ion mode for 240 min. Survey scans were acquired at a resolution of 70000 for the MS scan and 17500 for the MS/MS scan at m/z 200, with the isolation window of 1.6 *m/z*. The maximum ion injection times were 10 and 60 ms for MS and MS/MS, respectively, and the automatic gain control (AGC) target was set to 3E6. Other parameters were as follows: dynamic exclusion 40 s, underfill ratio 0.1%, and collision energy 30 eV.

The raw data of MS/MS spectra were processed using Mascot 2.2 and Proteome Discoverer 1.4 for database search and quantitative analysis. The MASCOT parameters were set as follows: peptide mass tolerance, 20 ppm; fragment mass tolerance, 0.1 Da; max missed cleavages, 2. All data were based on at least one unique peptide with 99% confidence, and protein identification was determined by false discovery rate (FDR) ≤1% as the screen threshold for peptide after mass spectrometric analysis and database Mascot search^45^.

### Multiple reaction monitoring (MRM) quantification of selected differential proteins

According to the iTRAQ results, several key differentially expressed proteins (see Table S1, Supplementary information) were selected and quantified by multiple reaction monitoring (MRM). For MRM analysis, the basic requirements of selected peptides were as follows: 10–15 amino acids length, unique peptide of differential proteins, no posttranslational protein modification (such as methionine oxidation and cysteine alkylation), and high count numbers in the iTRAQ tests. The protein translation initiation factor IF-2 was used as the internal standard. In brief, after the separation by HPLC, MS analysis of samples were conducted using AB SCIEX QTRAP 5500 system interfaced with the Nanospray III source and triplicate LC-MS/MS runs were performed for each sample. The parameters for MS analysis were as below: positive ion mode for 60 min, mass range of 100–1000 *m/z*, and mass tolerance 250 mDa. Raw quantitation data were confirmed by AB SCIEX ProteinPilot 4.5 software with the restriction of confidence >0.95, and the peptide transition intensity was analyzed quantitatively by software Skyline 2.5.0 (University of Washington). Triplicated biological samples were used in the MRM quantification test, and the differential expressed proteins were considered to be significantly regulated compared to the control when the *p* < 0.05.

### Denitrifying enzyme activity measurement

For the enzymatic activity measurement, the crude cell extracts were prepared firstly. Cells were harvested by centrifugation at 5000 rpm for 10 min, washed twice with 100 mM PBS (pH 7.4), and resuspended in the same buffer. The suspension was disrupted by sonication at 20 KHz for 5 min, and then the cell debris was removed by centrifugation at 12000 rpm for 10 min. The prepared crude cell extracts were immediately used for the determination of specific enzyme activities. All the above operations were carried out at 4 °C. The enzymes activities were based on protein content, and the content of protein was determined by the method of Lowry *et al.* with bovine serum albumin as standard^43^.

The assay mixture for reactions contained 10 mM PBS buffer (pH 7.4), 1 mM methyl viologen, 5 mM Na_2_S_2_O_4_, and 1 mM reaction substrate (KNO_3_, NaNO_2_, NO or N_2_O). The reaction was started by adding 0.3 mL crude cell extracts, and the mixture was immediately settled in a 30 °C incubator. The data were collected every 10 min on a spectrophotometer (for NAR and NIR, Shimazu UV1800, Japan) or microsensors (for NOR and N_2_OR, Unisense, Denmark). Finally, their activities were calculated as μmol substrate/(min·mg protein).

### Intracellular iron detection

According to literatures, intracellular iron was determined by fluorescence probe calcein-AM (calcein-acetomethoxy)^46^,^47^. The permeant calcein-AM can enter cells, bind to intracellular iron and exist in the iron-binding form [CA-Fe], which emits fluorescence at 488 nm excitation. Firstly, the bacterial cells were obtained by centrifuging at 3500 rpm for 10 min, and then washed by HEPES (N-2-hydroxyethylpiperazine-N-2 -ethanesulfonic acid) buffer (20 mM HEPES, 153 mM NaCl, 5 mM KCl, 5 mM glucose, pH 7.4) for three times. For dye labeling, the cells were adjusted to 1 × 10^7^ cells/mL in HEPES buffer with 0.5 μM calcein-AM for 30 min incubation at 37 °C. Then, the cells were rinsed with HEPES buffer to remove the extracellular calcein-AM and then loaded in a 96-well plate with the same buffer for fluorescence detection. Flow cytometric analysis was performed with a BD Accuri C6 (USA) using a 488 nm argon ion laser, and fluorescence emissions were passed through a 530/30 nm wideband filter. During the detection process, 10000 cells were collected and the fluorescence intensities were measured for analysis.

### Analytical methods

The concentrations of NO_3_^−^ and NO_2_^−^ were determined by a spectrophotometer according to the Standard Methods^48^, and glucose was measured by anthrone-H_2_SO_4_ method^49^. The determination of N_2_O was conducted by a gas chromatograph (GC) (Agilent 7820A, USA) with an electron capture detector (ECD). The N_2_O in gas was directly sampled and injected into the sample inlet of GC by a syringe, and the N_2_O dissolved in aqueous solution was detected after using headspace with equilibrium temperature and time of 25 °C and 3 h, respectively. The optical density (OD_600_) of bacterial suspension was measured using a spectrophotometer (Shimadzu UV1800, Japan) at 600 nm every 4 h.

### LDH release and cell viability

The LDH release assay was used to determine the membrane integrity for intracellular LDH would release to the extracellular media if the cellular membrane was damaged^31^. The Cytotoxicity Detection Kit was purchased from Roche Applied Science and the reaction mixture was prepared according to the manufacturer’s instructions. After exposed to CuO NPs for 24 h, the culture supernatants were obtained by centrifugation (12000 rpm for 3 min) and seeded in a 96-well plate, and then 50 μL of reaction mixture was added for 30 min incubation at 30 °C. Finally, the data were obtained at 490 nm absorbance via a microplate reader (BioTek, USA). Cell viability was detected by Cell Counting Kit-8 (CCK-8, Dojindo) according to the manufacturer’s instructions. In detail, 100 μL of the cell suspensions were obtained after 24 h exposure and placed in a 96-well plate, following by seeding 10 μL of CCK-8 for 30 min at 30 °C. Thereafter, the absorbance was recorded at 450 nm via a microplate reader (BioTek, USA).

### SEM and TEM

SEM images were used to obtain the surface morphology of bacteria treated by CuO NPs, and the procedure of samples preparation was documented in literatures^50^,^51^,^52^. After 24 h exposure to CuO NPs, bacterial cells were fixed with 2.5% glutaraldehyde for 2 h, washed 3 times with 0.1 M phosphate buffer solution (PBS, pH 7.4), and then dried in a vacuum freeze dryer. Finally, the dried samples were sputter coated with gold and then viewed under a FEI Quanta 200 SEM at 20 kV equipped with energy dispersive spectroscopy (EDS). TEM was employed to investigate the intracellular presence of CuO NPs, and the samples were prepared according to literatures^16^,^53^. The treated bacteria were fixed with 2.5% glutaraldehyde for 2 h, postfixed in 1% osmic acid for 1 h, washed with 0.1 M phosphate buffer solution (pH 7.4) for 3 times, and dehydrated in increasing concentration of acetone (30%, 50%, 70%, 90% and 100%) for 20 min each time. Then, the samples were permeated and impregnated by pure resin for 5 h at 60 °C, and 60 nm ultrathin sections were made for subsequent imaging by a Tecnai G2 (FEI, USA) with 120 kV accelerating voltage.

### Intracellular NADH detection

The measurement of intracellular NADH was performed according to the literature^54^. The samples were obtained by centrifugation at 15000 rpm for 1 min, and 300 μL of 0.2 M NaOH was added to re-suspend the pellets. Then, the samples were placed in 50 °C water bath for 10 min, cooled down to 0 °C on ice, and neutralized by adding 300 μL of 0.1 M HCl dropwise while vortexing. After the centrifugation at 15000 rpm for 5 min, supernatants were used for the following measurement. The assay mixture contained equal volumes of 1.0 M Bicine buffer (pH 8.0), ethanol, 40 mM EDTA (pH 8.0), 4.2 mM thiazolyl blue (MTT), and twice the volume of 16.6 mM phenazine ethosulfate (PES), and then incubated at 30 °C for 10 min. The reaction mixture was prepared by following volumes: 50 μL neutralized cell extract, 0.3 mL distilled water, and 0.6 mL assay mixture. The reaction was started by adding 50 μL of alcohol dehydrogenase (ADH, 500 U/mL), and then the absorbance at 570 nm was recorded at 30 °C. The concentrations of NADH were calibrated with standard solutions of NADH, and the final intracellular NADH levels were calculated based on protein content.

### Statistical analysis

All tests were performed in triplicate, and the results were expressed as mean ± standard deviation. An analysis of variance (ANOVA) was used to test the significance of results and *p* < 0.05 was considered to be statistically significant.

## Additional Information

**How to cite this article**: Su, Y. *et al.* Alteration of intracellular protein expressions as a key mechanism of the deterioration of bacterial denitrification caused by copper oxide nanoparticles. *Sci. Rep.* **5**, 15824; doi: 10.1038/srep15824 (2015).

## Supplementary Material

Supplementary Information
