# Supplementary material for: Alteration of intracellular protein expressions as a key mechanism of the deterioration of bacterial denitrification caused by copper oxide nanoparticles
Source: Sci Rep. 2015 Oct 28;5:15824. doi: 10.1038/srep15824 (PMC4623765; doi:10.1038/srep15824)
Supplement: Supplementary Information [file srep15824-s1.pdf]

Supplementary Information

Alteration of intracellular protein expressions as a key mechanism of the deterioration of bacterial denitrification caused by copper oxide nanoparticles

Yinglong Su, Xiong Zheng*, Yinguang Chen*, Mu Li, Kun Liu

(*State Key Laboratory of Pollution Control and Resource Reuse, School of Environmental Science and Engineering, Tongji University, 1239 Siping Road, Shanghai 200092, China*)

*Corresponding author

Tel.: +86 21 65981263

Fax: +86 21 65986313

E-mail: yg2chen@yahoo.com (Y.C.); xiongzheng@tongji.edu.cn (X.Z.)

Supplementary Methods

Copper Ion Dissolution from CuO NPs and Cu^{2+} Toxicity Test. To determine the released Cu^{2+} from CuO NPs, 0.05, 0.1 and 0.25 mg/L CuO NPs were prepared in the mineral media according to the similar procedure of the exposure experiment, and the only difference was the absence of bacteria in media. After settled in the shaker, the nanoparticles suspensions were gotten and then centrifuged at 14000 rpm for 10 min, and the supernatant was taken for dissolved copper ions detection by inductively coupled plasma mass spectrometry (ICP-MS, Agilent Technologies, Santa Clara, CA). After the determination of dissolved Cu^{2+}, the ion toxicity test was conducted. Denitrifying bacteria were exposed to corresponding Cu^{2+} in media, and the concentrations of NO_3^--N, NO_2^--N and N_2O were measured every 4 h. After 24 h exposure, the cell viability and denitrifying enzyme activities were determined by respective assays. All the procedures were similar to the CuO NPs toxicity test.

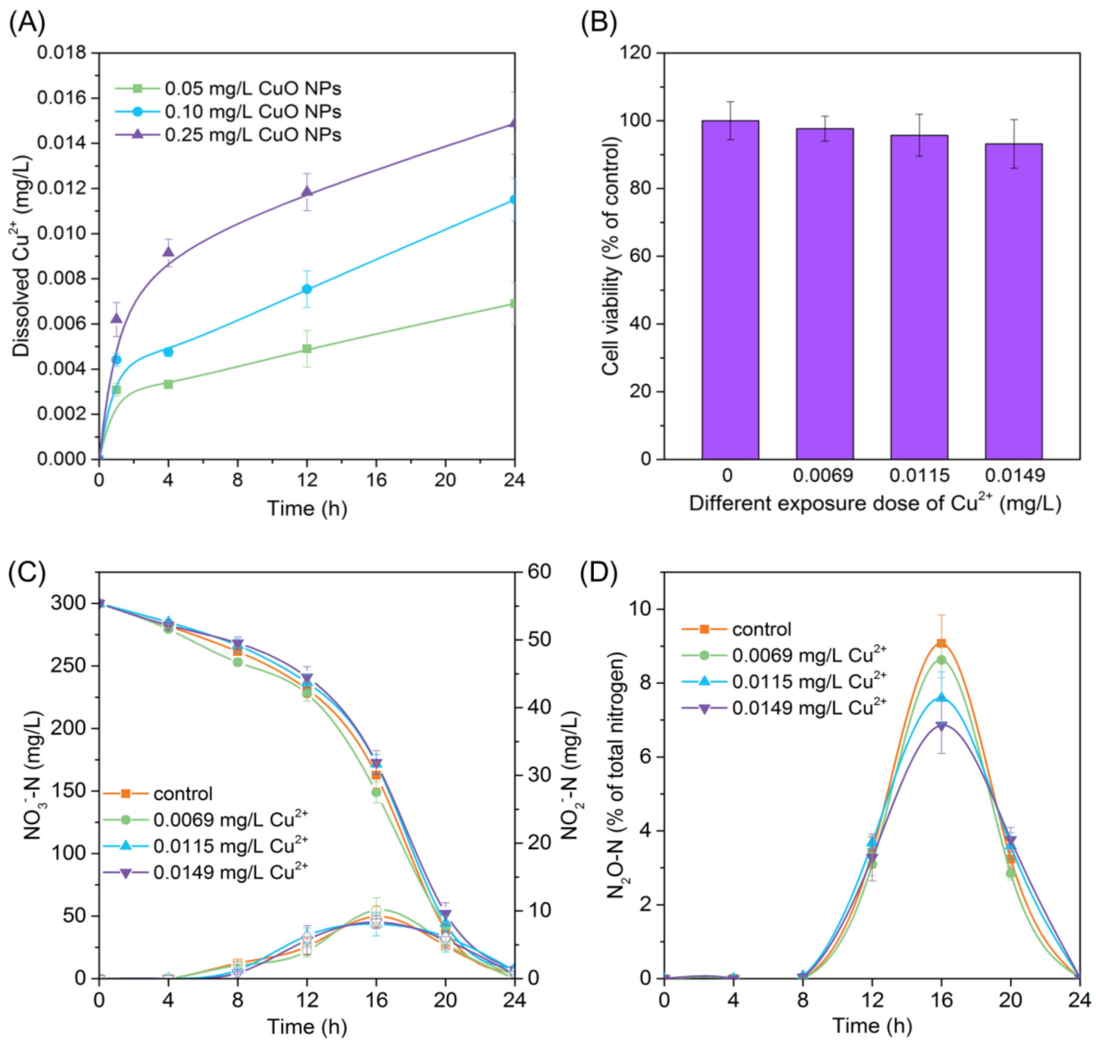

Figure S1. Dissolution of copper ion from CuO NPs at different concentrations (A); The potential effects of dissolved Cu^{2+} on nitrate removal efficiency and cell viability of *P. denitrificans* after 24 h exposure (B); Effects of Cu^{2+} on the variations of NO_3^--N (solid, C), NO_2^--N (hollow, C) and N_2O-N (D) during 24 h exposure tests. Error bars represent standard deviations of triplicate measurements.

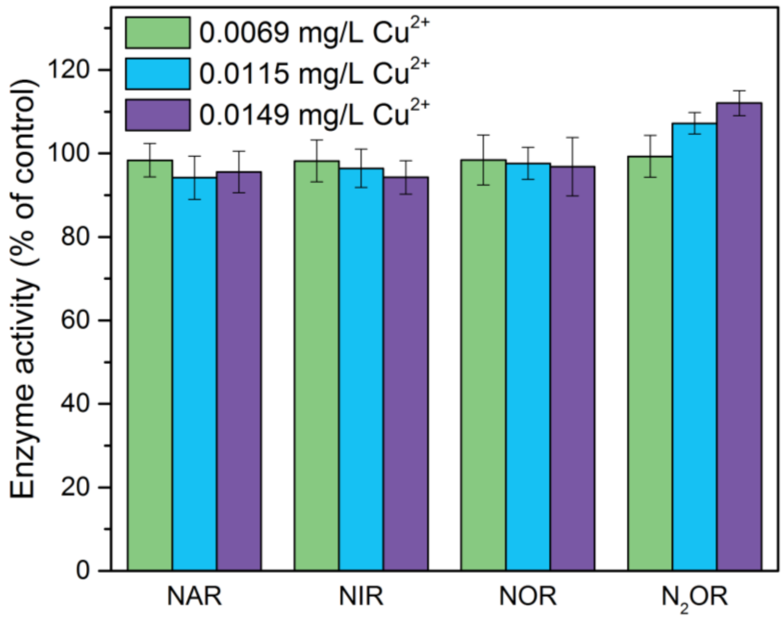

Figure S2. The effects of Cu^{2+} exposure on the activities of NAR, NIR, NOR, and N_2OR at time of 24 h. Error bars represent standard deviations of triplicate measurements

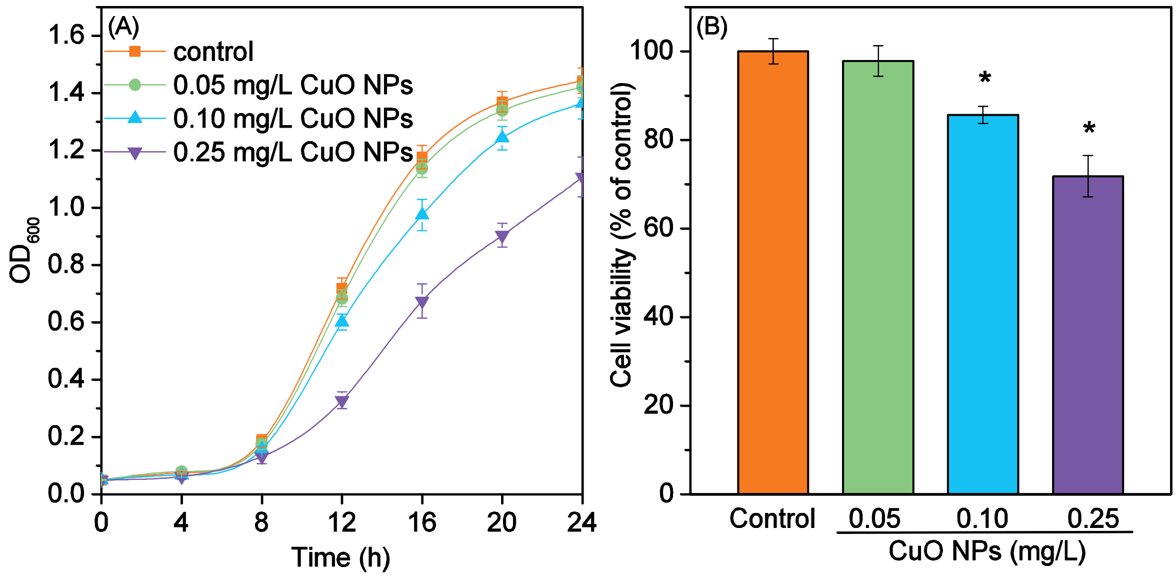

Figure S3. The growth of *Paracoccus denitrificans* exposed to different dose CuO NPs (A), and the relative viability of *P. denitrificans* after 24 h of CuO NPs exposure (B).

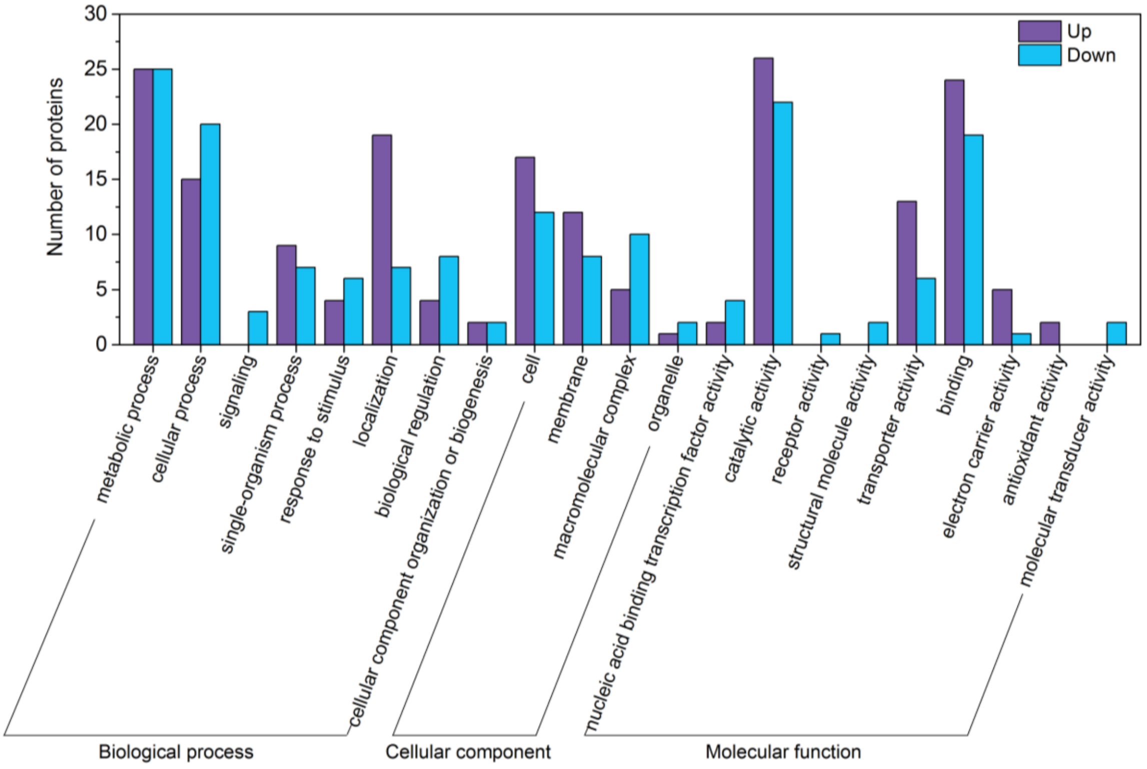

Figure S4. Classification of identified differential proteins of *P. denitrificans* with or without CuO NPs exposure according to gene ontology annotation in molecular function, cellular components and biological processes. The bars in figure represent the ratio of protein differences between the CuO NPs treatment and control.

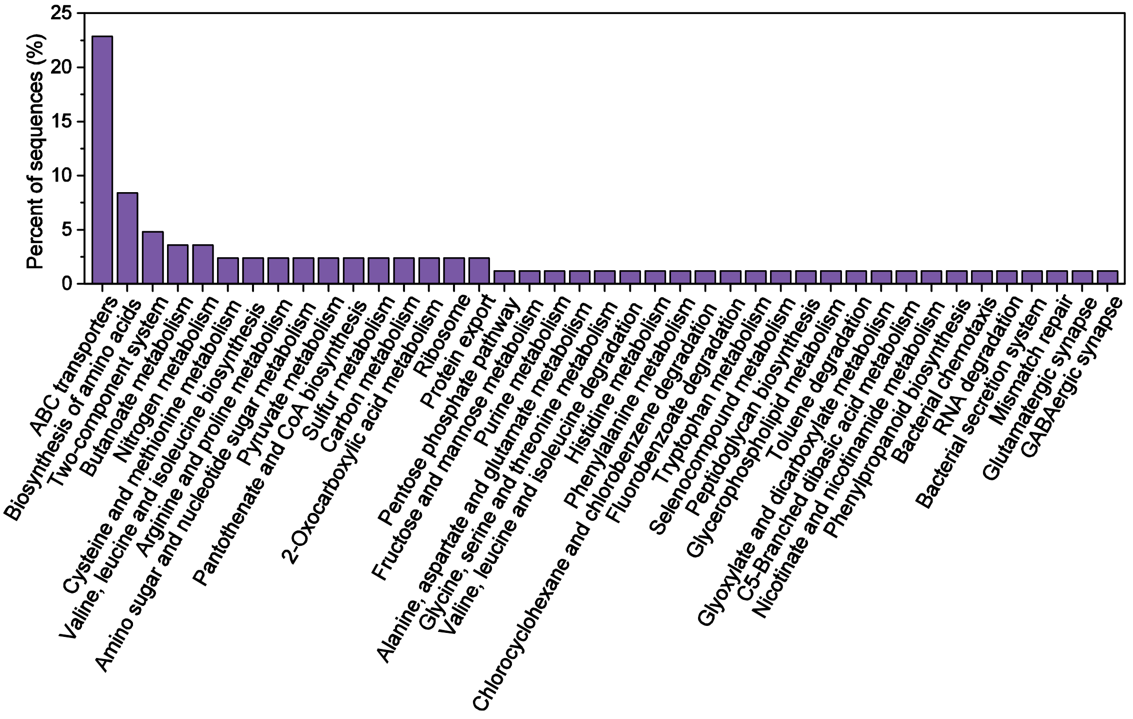

Figure S5. Functional classification of differentially expressed proteins of *P. denitrificans* under CuO NPs stress. The classification is based on KEGG (http://www.kegg.jp/kegg/pathway.html).

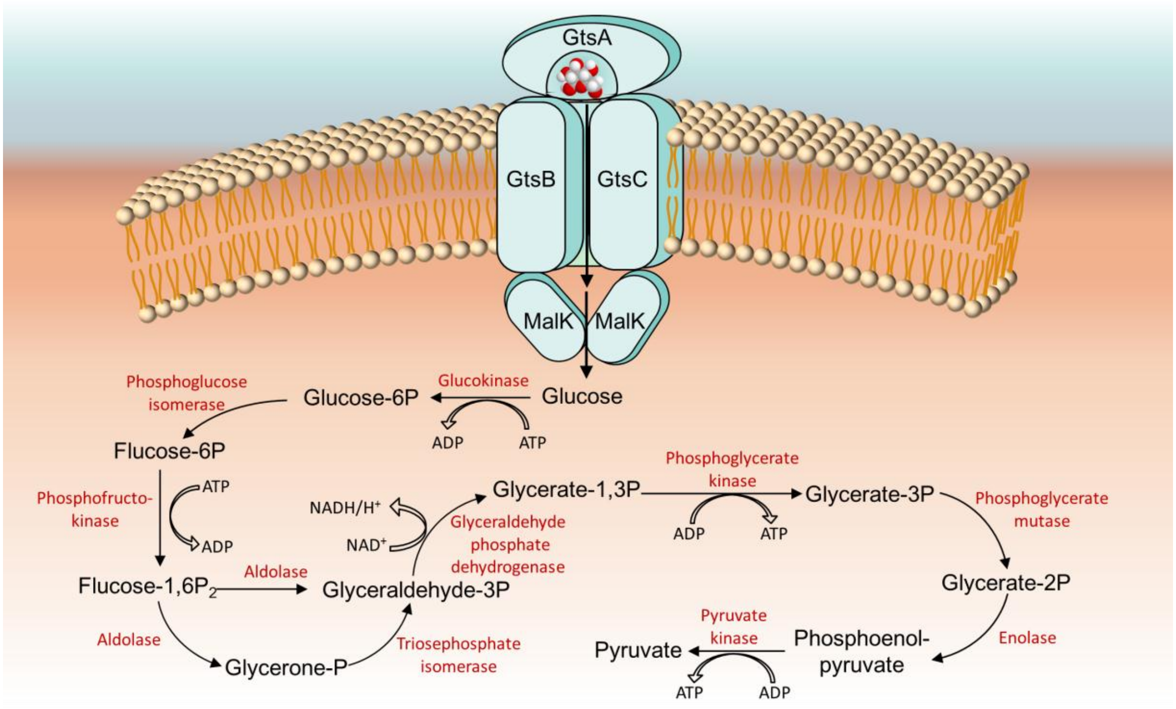

Figure S6. The schematic diagram of transport and intracellular metabolism of glucose.

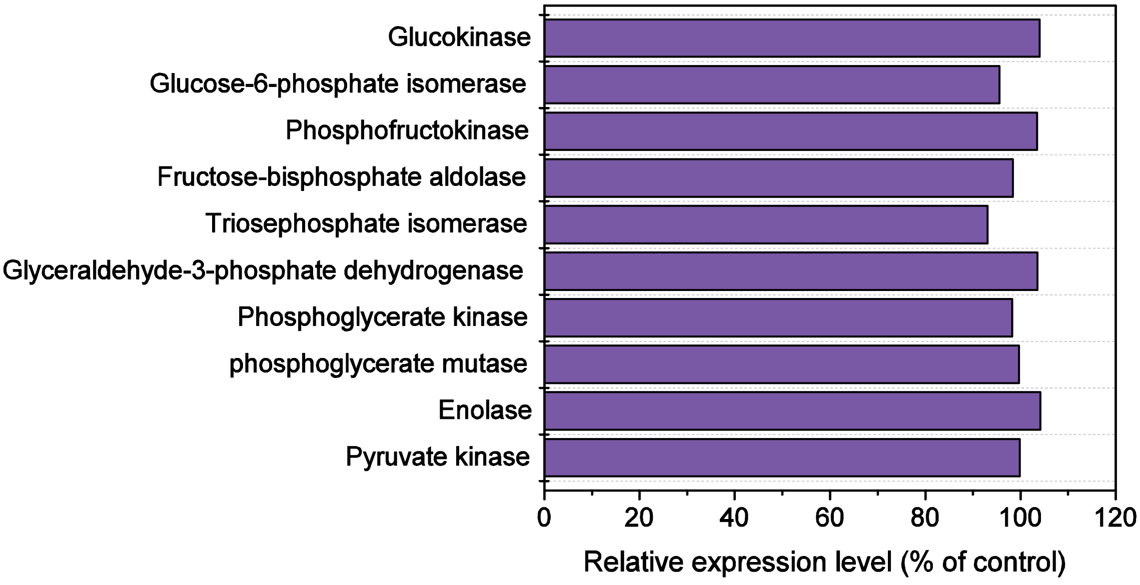

Figure S7. Effect of 0.25 mg/L CuO NPs on the relative expressions of proteins involved in glycolysis process.

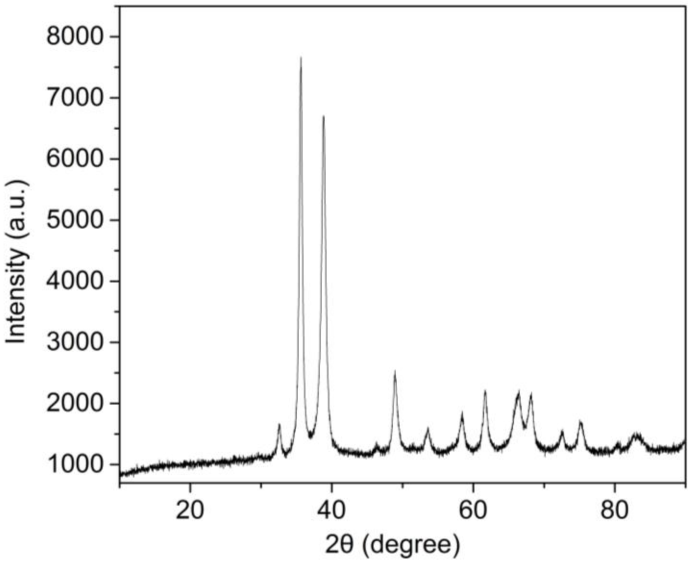

Figure S8. X-ray diffraction (XRD) pattern of CuO NPs.

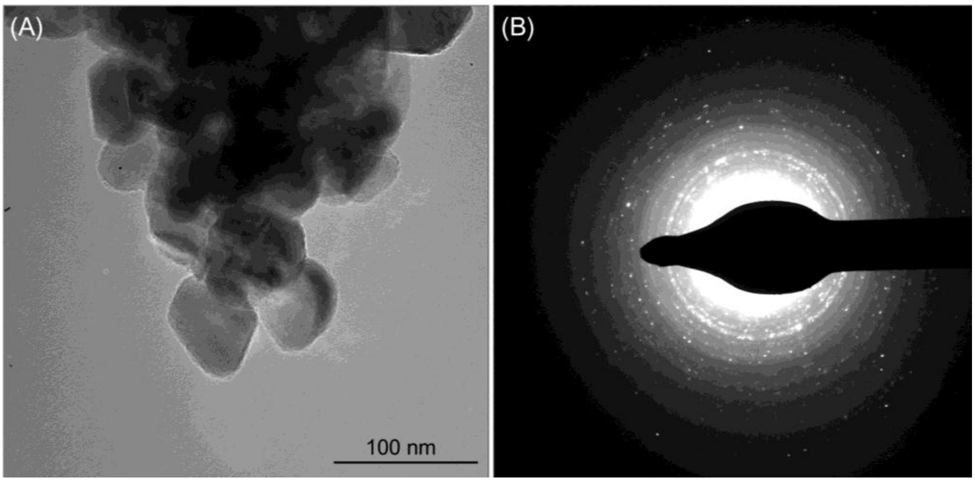

Figure S9. TEM images of CuO NPs (A) and the selected area electron diffraction (SAED) pattern (B) of the nanoparticles in A. These images confirmed the CuO NPs and provided the diameter.

Table S1. Relative expressions of differential proteins induced by 0.25 mg/L CuO NPs and the corresponding sequences of peptides in the MRM quantification.

Protein accession	Protein name	Relative expression [a]	p value	Sequences of peptides in the MRM quantification
A1B7I2	GtsB, Carbohydrate ABC transporter membrane protein 1	0.80	0.019	AAQVDGIPTHR<br>YEFEGIGQYER
A1B7I1	GtsA, Carbohydrate ABC transporter substrate-binding protein	1.057	0.072	GYVDDNFSGR<br>VLSGNAPTAVQLK
A1B7I4	MalK, Carbohydrate ABC transporter ATP-binding protein	0.949	0.067	EGAAPLVITDPVAR<br>QLQIEPLLAR
A1B7I3	GtsC, Carbohydrate ABC transporter membrane protein 2	0.923	0.058	TGSLLSLPR<br>NYYVTIPQELVR
A1B6B9	FhuD, Periplasmic binding protein	0.606	0.002	ELAQIFDVEER<br>EPQLVTTQFEFHVGPQGAVGTR
A1B9V6	Respiratory nitrate reductase alpha subunit	0.847	0.026	DYDADVPFTPAWAER<br>GGPVVWISEIDAR
A1B9V5	Respiratory nitrate reductase beta subunit	0.825	0.013	EIVEDAYDLR<br>TLDGVVNLGVAK
Q51700	Nitrite reductase	0.708	0.001	DQESALVVVDDK<br>SVLDTGYAVHISR
A1B9T9	Nitrous-oxide reductase	1.67	0.003	AQAEADGVDIDNWTEEVIR<br>LSPTVTVLDVTR<br>SAVVAEPELGLGPLHTAFDGR
A1B311	Cytochrome c, class I	0.535	0.011	AHGGDWTPEALQEFLTNPK
P13627	Cytochrome c1	0.844	0.041	TLADEGGPQLPEDQVR
P05417	Ubiquinol-cytochrome c reductase iron-sulfur subunit	0.857	0.035	EVDLGQLIDR<br>GPAPQNLHIPVAEFLDDTTIK
A1BAG3	Electron transport protein SCO1/SenC	0.66	0.022	LTATDGTEFSQAALK<br>TGSGSGSVADAGAAALGR<br>VFFVTVDPER
P38974	Electron transfer flavoprotein subunit alpha	0.886	0.025	AAVDSGYAPNDWQVGQTGK<br>ESFAIIEELADK
A1AZY8	NADH: flavin oxidoreductase/NADH oxidase	0.788	0.044	FPLEVFEAVR<br>LFEPIAIGGQTLANR

[a] It was calculated by dividing the protein expression exposed to 0.25 mg/L CuO NPs by that in the control.
